# Atractylenolide II reverses the influence of lncRNA XIST/miR‐30a‐5p/ROR1 axis on chemo‐resistance of colorectal cancer cells

**DOI:** 10.1111/jcmm.14148

**Published:** 2019-03-25

**Authors:** Ruijuan Zhang, Zhijun Wang, Qianyun Yu, Jun Shen, Wenji He, Dongqing Zhou, Qingqing Yu, Jiawei Fan, Shurong Gao, Lihong Duan

**Affiliations:** ^1^ Department of Traditional Chinese Medicine Putuo People's Hospital Affiliated to Tongji University Shanghai China; ^2^ Department of Traditional Chinese Medicine Wuliqiao Community Health Center of Huangpu District Shanghai China; ^3^ Department of Rheumatology Putuo People's Hospital Affiliated to Tongji University Shanghai China

**Keywords:** atractylenolide, cell proliferation, cell viability, chemoresistance, colorectal cancer, lncRNA XIST, miR‐30a‐5p, ROR1

## Abstract

This investigation was conducted to elucidate whether atractylenolide II could reverse the role of lncRNA XIST/miR‐30a‐5p/ROR1 axis in modulating chemosensitivity of colorectal cancer cells. We totally collected 294 pairs of colorectal cancer tissues and adjacent normal tissues and also purchased colorectal cancer cell lines and human embryonic kidney cell line. 5‐fluorouracil, cisplatin, mitomycin and adriamycin were designated as the chemotherapies for colorectal cell lines, and atractylenolides were arranged as the Chinese drug. The expressions of XIST, miR‐30a‐5p and ROR1 were quantified with aid of qRT‐PCR or Western blot, and luciferase reporter gene assay was implemented to determine the relationships among XIST, miR‐30a‐5p and ROR1. Our results demonstrated that XIST and ROR1 expressions were dramatically up‐regulated, yet miR‐30a‐5p expression was down‐regulated within colorectal cancer tissues (*P* < 0.05). The overexpressed XIST and ROR1, as well as under‐expressed miR‐30a‐5p, were inclined to promote viability and proliferation of colorectal cells under the influence of chemo drugs (*P* < 0.05). In addition, XIST could directly target miR‐30a‐5p, and ROR1 acted as the targeted molecule of miR‐30a‐5p. Interestingly, atractylenolides not only switched the expressions of XIST, miR‐30a‐5p and ROR1 within colorectal cancer cells but also significantly intensified the chemosensitivity of colorectal cancer cells (*P *< 0.05). Finally, atractylenolide II was discovered to slow down the viability and proliferation of colorectal cancer cells (*P *< 0.05). In conclusion, the XIST/miR‐30a‐5p/ROR1 axis could be deemed as pivotal markers underlying colorectal cancer, and administration of atractylenolide II might improve the chemotherapeutic efficacy for colorectal cancer.

## 1 INTRODUCTION

Colorectal cancer (CRC), a malignancy stemming from epithelium or gland of colorectal mucosa, stood as the third most common cancer around the world.^1^ The worldwide prevalence of new CRC cases has risen swiftly to 1.2 million per year, with approximately 0.5 million deaths per year.^2^ The hazard factors for CRC were acknowledged as family history, inflammatory bowel disease, smoking, excessive consumption of alcohol, intake of substantial red meat, obesity and diabetes. For CRC patients of stage I‐II, surgery was prioritized without risky parameters,^3^ while the patients of advanced CRC were still faced with neoplastic recurrence and metastasis that was accompanied by dramatically reduced 5‐year survival.^4^ Furthermore, the appearance of drug resistance confined the efficacy of chemotherapies and even led to chemotherapeutic failure. Thus, how to seek for novel biomarkers for early‐stage CRC and enhance chemosensitivity of CRC has been increasingly pivotal.

Accumulating investigations have documented that expressions of lncRNAs metastasis‐associated lung adenocarcinoma transcript 1 (MALAT1), HOX antisense intergenic RNA (HOTAIR), colon cancer‐associated transcript 1 (CCAT1), colorectal neoplasia differentially expressed (CRNDE) and X‐inactive specific transcript (XIST) were elevated with aggravation of CRC, whereas lncRNAs maternally expressed gene 3 (MEG3) and RP11‐462C24.1 were lowly expressed when CRC became invasive and metastatic.^5^, ^6^, ^7^, ^8^, ^9^, ^10^, ^11^ Among them, the XIST was a principal gene for modulating inactivation of X chromosome within mammals, and it could merely be transcribed from inactivated X chromosome.^12^ This lncRNA mattered much for neoplastic pathologies, because of its responsibility for altering genetic expressions by affecting the stability of heterochromatins.^13^ For instance, XIST was indispensable for maintaining the long‐term survival of hematopoietic stem cells,^14^ and knock‐down of XIST restrained growth and metastasis of glioma cells.^15^ Besides, the up‐regulated expression of XIST tended to differentiate NSCLC tissues from normal tissues,^16^ and the receiver operating characteristic curve suggested a highly diagnostic value of XIST for NSCLC sufferers.^17^ Despite the carcinogenic predisposition of XIST, the specific molecular network of XIST that contributed to CRC development remained far from complete.

With starbase software (version 2.0) adopted to herald the target miRNAs of XIST,^18^ the predicted miR‐30a has been reported to obviously weaken the progression of multiple neoplasms, including NSCLC, hepatic carcinoma, pancreatic cancer and melanoma.^19^, ^20^, ^21^ Additionally, miR‐30a was also validated to symbolize the poor prognosis of CRC patients, when it was down‐regulated.^22^ Further in vivo and in vitro experiments also argued that miR‐30a could prohibit migration and invasion of cancer cells by regulation of downstream signalling pathways or target genes.^23^, ^24^, ^25^ Taking receptor‐tyrosine‐kinase‐like orphan receptor 1 (ROR1) for instance, its high expression appeared positively correlated with malignant traits and poor prognosis of CRC patients,^26^ and silencing of it could remarkably delay the invasive and migratory pace of chemoresistant cancer cells.^27^ To sum up, miR‐30a‐modifying ROR1 might be involved in the aetiology of diverse neoplasms, yet whether this combined action could act on progression and chemosensitivity of CRC required further researches.

As already reported, combined treatments of traditional Chinese medicine (TCM) and chemotherapy for post‐operative cancer patients not merely relieved the toxicity and resistance generated because of chemotherapy but also raised the patients’ own immunity.^28^, ^29^, ^30^, ^31^ Within this study, white atractylodes rhizome, originated from a perennial herb that pertained to atractylodes macrocephala koidz, was adopted as the candidate TCM treatment regimen, with ingredients featured by anti‐inflammatory and anti‐neoplastic functions.^32^ For instance, atractylenolide I (AO‐I)^33^ could fight against tumours by inducing apoptosis of leukaemia cells and it acted against inflammation partly by bringing down levels of IL‐1, TNF‐α and proteolysis‐inducing factor.^34^, ^35^ Nevertheless, few investigations were carried out to explore whether AO would attenuate chemo‐resistance in cancers, especially CRC, through the modification of certain signalling pathways. In response, this investigation was intended to validate the role of XIST/miR‐30a/ROR1 axis and AO in regulating the chemosensitivity of CRC cells.

## 2 METHODS AND MATERIALS

### 2.1 Study samples

We collected 294 pairs of CRC tissues and paracarcinoma tissues (>5 cm distant from cancer tissues) from CRC patients who underwent surgical removal in the Putuo People's Hospital Affiliated to Tongji University from March 2012 to October 2013. The patients all satisfied the following criteria: (a) they were aged ≥18 years old with all‐sided individual information; (b) they were histopathologically examined as CRC; (c) they accepted CRC treatments for the first time; (d) they showed no sign of other malignancies or serious disorders and underwent no other surgery or chemotherapies; (e) their Karnofsky performance status (KPS) score was ≥70; and (f) they were without serious cardiopulmonary dysfunction or surgical contraindications. Meanwhile, the candidates would be rejected if: (a) they failed to provide any obvious pathologic findings; (b) their clinical information was incomplete; (c) they have been treated with chemotherapies; and (d) their KPS score was <70. The included CRC patients were staged in line with the criteria formulated by American Joint Committee on Cancer/Union for International Cancer Control (AJCC/UICC). Moreover, the procedures of this study have acquired the approval of Putuo People's Hospital Affiliated to Tongji University and the ethics committee of Putuo People's Hospital Affiliated to Tongji University, and all patients involved have signed informed consents.

### 2.2 Follow‐up survey

The CRC patients were surveyed in the form of outpatient review and phone call during the follow‐up period (ie 5 years). Immediately after surgery, the subjects were strictly monitored every 3‐6 months. The inspection items consisted of: (a) physical examination, especially anal examination; (b) examination of blood carcinoembryonic antigen and indicators that have ever increased; (c) X‐ray inspection for lung, B‐mode ultrasound for abdomen and pelvic cavity, as well as magnetic resonance imaging for brain; and (d) enhanced computed tomography (CT) examination for chest pelvic, enteroscopy and whole body bone scan per year. Local recurrence and distant metastasis were determined when any of liver, lung, bone and brain showed metastatic symptoms.

### 2.3 Cell culture

The purchased CRC cell lines (ie SW480, HCT116, Lovo and SW620; Shanghai Institutes for Biological Sciences, China) and human embryonic kidney cells (HEK293T; American Type Culture Collection, Manassas, VA, USA) were cultured within RPMI medium (Gibco, Grand Island, NY, USA) that contained 100 mL/L foetal bovine serum in 5% CO_2_ and saturated humidity at 37°C. The nutrient solution was changed every 2‐3 days, and cells at the logarithmic phase were digested by pancreatin and passaged before performing the following experiments.

### 2.4 Cell transfection

Cells at the density of 5 × 10^4^/well were inoculated into 24‐well plates, with the culture medium free from antibiotics. When cells grew to 70% confluence, pcDNA‐XIST (Genechem, Shanghai, China), si‐XIST‐1 (sense: 5′‐GCAAAUGAAAGCUACCAAU‐3′, antisense: 5′‐AUUGGUAGCUUUCAUUUGC‐3′, Genechem, Shanghai, China), si‐XIST‐2 (sense: 5′‐GCACAAUAUCUUUGAACUA‐3′, antisense: 5′‐UAGUUCAAAGAUAUUGUGC‐3′, Genechem, Shanghai, China), si‐XIST‐3 (sense: 5′‐CUAGAAUCCUAAAGGCAAA3′, antisense: 5′UUUGCCUUUAGGAUUCUAG3′, Genechem, Shanghai, China), miR‐30a‐5p mimic (sense: 5′‐UGUAAACAUCCUCGACUGGAAG‐3′, antisense: 5′‐CUUCCAGUCGAGGAUGUUUACA‐3′, Ribobio, Guangzhou, China), miR‐30a‐5p inhibitor (sense: 5′‐CUUCCAGUCGAGGAUGUUUACA‐3′, anti‐sense: 5′‐UGUAAACAUCCUCGACUGGAAG‐3′, Ribobio, Guangzhou, China), pCMV6‐ROR1 (OriGene Technologies, Rockville, MD, USA), and si‐ROR1 (sense: 5′‐AUCCGGAUUGGAAUUCCCAUG‐3′, antisense: 5′‐CAUGGGAAUUCCAAUCCGGAU‐3′; sense: 5′‐CUUUACUAGGAGACGCCAAUA‐3′, anti‐sense: 5′‐UAUUGGCGUCUCCUAGUAAAG‐3′, Open Biosystem, Huntsville, AL, USA) were transfected into Lovo cells, according to the instructions of Lipofectamine3000 (Invitrogen, Carlsbad, CA, USA).

### 2.5 Administration of AO to cells

Cells were seeded into 96‐well plates at the density of 1 × 104 per well for 24 hours. Subsequently, AO‐I (Lot. No.: MUST‐13012005, Chengdu Institute of Biology affiliated to Chinese Academy of Sciences, China), AO‐II (Lot. No.: MUST‐13012006) and AO‐III (Lot. No.: MUST‐13012007) at the concentrations of 200, 100, 50, 25, 12.5 and 0 μg/mL were, respectively, managed to treat the cells. The AO with the optimal dosage was chosen for conduction of following time‐effect experiments.

### 2.6 Evaluation of chemosensitivity with MTT assay

Cells growing at the logarithmic phase were inoculated into 96‐well plates at the density of 1 × 10^4^/mL. The cells were respectively administrated with 5‐fluorouracil (5, 10, 20, 40 and 80 μg/mL), cisplatin (5, 10, 25, 50 and 100 μg/mL), mitomycin (1, 5, 10, 15 and 30 μg/mL) and adriamycin (2.5, 5, 10, 20 and 40 μmol/L). Meanwhile, cells without addition of drugs were designated as the control group. When cells in the control group reached 90% confluence, 20 μL MTT solution (5 mg/mL) (Amresco, Solon, OH, USA) was added to each well for at least 4‐hour cultivation. Then, medium was discarded, and 150 μL dimethyl sulphoxide (Genebase, Guangzhou, China) was supplemented for 15‐minute shaking. The absorbance values at the wavelength of 490 nm (A490) were determined using a microplate reader (model number: ELX800UV, USA), and the half maximal inhibitory concentration (IC50) values were also calculated.

### 2.7 Colony formation assay

Cells at the logarithmic phase were seeded into 6‐well plates at a concentration of 400‐500 per well, and they were cultivated in 5% CO_2_ at 37°C for 2‐3 weeks. The culture would be terminated when clonal cell cluster became visible by naked eye. After discarding the supernatants and rinsing the cells with phosphate buffer (PBS) for twice, the cells were fixed with 4% paraformaldehyde for 20 minutes. When the stationary liquid was removed, we added crystal violet to dye them for 30 minutes. The number of colonies with >50 cells was counted carefully under the optical microscope. The colony formation rate (%) was the result of number of colonies divided by the number of cells seeded.

### 2.8 Cell apoptosis assay

The cells in each treatment group were digested with 0.25% pancreatin and were then washed with pre‐cooled PBS at 4°C. Exactly 250 μL binding buffer was supplemented to resuspend the cells, and 100 μL (about 1 × 10^5^ cells) therein was supplemented with 5 μL Annexin V‐fluorescein isothiocyanate and 10 μL propidium iodide (PI) (20 μg/mL) (Beyotime, Shanghai, China) successively for 15‐minute staining at room temperature. Then, the reaction products were detected on the flow cytometry (model: FACScan; Becton‐Dickinson, Franklin Lakes, NJ, USA).

### 2.9 RNA extraction and quantitative real‐time polymerase chain reaction (qRT‐PCR)

Total RNA was extracted by applying Trizol method (Life Technologies, Gaithersburg, MD, USA), and electrophoresis with 1% agarose gel was performed to detect the purity and integrity of RNA under the ultraviolet lamp. Besides, the concentration of RNA was measured with usage of micro‐spectrophotometer (model: Nano‐100; ThermoFisher, Waltham, MA, USA), and the samples with A260/A280 ratio of 1.8‐2.0 were collected for reverse transcription. Following the instructions stipulated in the PrimeScript^®^RT reagent kit (TaKaRa, Tokyo, Japan), we reversely transcribed the RNAs into cDNAs. According to the manual of SYBR I Premix Ex Taq^™^ kit (TaKaRa), the PCR reactions were performed with aid of ABI 7500 real‐time PCR system (USA). Furthermore, Taqman^®^ MicroRNA Reverse Transcription Kit and Taqman^®^ Universal Master Mix II kit were employed to determine the expressions of miR‐30a‐5p. The GAPDH was taken as the internal reference for XIST and ROR1, and U6 was set as the internal reference for miR‐30a‐5p. The relative expressions of genes were normalized through comparative CT value method (ie 2^−△△Ct^ method), and the primers for XIST, miR‐30a‐5p and ROR 1 were enlisted in Table 1.

**Table 1:** Nucleic acid sequences of primers used for real‐time reverse‐transcriptase PCR

Gene primer	Nucleotide sequence (5′‐3′)
LncRNA XIST	F: ACG CTG CAT GTG TCC TTA G
	R: GAG CCT CTT ATA GCT GTT TG
ROR1	F: AAT GCA GAG TAA CGT GGA AGT GGT C
	R: TGG TCG CTC AAT CTC CAG GTC
GAPDH	F: AGA AGG CTG GGG CTC ATT TG
	R: AGG GGC CAT CCA CAG TCT TC
miR‐30a‐5p	F: ACT CAG CTG GTG TAA ACA TCC TCG AC
	R: TGG TGT CGT GGA GTC G
U6	F: CTC GCT TCG GCA GCA CA
	R: AAC GCT TCA CGA ATT TGC GT

F indicates a forward primer, and R indicates a reverse primer.

### 2.10 Western blotting

The tissues and cells were added with 100 μL lysis buffer RIPA (Beyotime, Shanghai, China), which were then centrifugated at 13053 g and 4°C for 15 minutes. The supernatants were then collected, and the concentration of total protein was determined through Bradford method. The protein lysate mixed with 5× loading buffer at the ratio of 5:1 was boiled for 5‐10 minutes. Subsequently, 20 μg protein mixed with 2× sodium dodecyl sulphate (SDS) loading buffer at the ratio of 1:1 was heated at 95°C for 10 minutes and then frozen for 2 minutes. Subsequently, 12% SDS‐polyacrylamide gel electrophoresis (PAGE) was performed, and the proteins on the PAGE gel were then electro‐transferred onto the polyvinylidene fluoride (PVDF) membrane. After incubating the PVDF membrane with blocking buffer for 1 hour, rabbit anti‐human primary antibodies (Abcam, Cambridge, MA, USA) against ROR1 (1:50, Cat. No: ab135669), Ki‐67 (1:5000, Cat. No: ab92742), PCNA (1:1000, Cat. No: ab152112) and GAPDH (1:2500, Cat. No: ab9485) were added for overnight inoculation of cells at 4°C. After washing the membrane with Tris‐buffered saline for 3 times, goat anti‐rabbit secondary antibodies (1:5000, Cat. No: ab97080; Abcam, Cambridge, MA, USA) marked with horseradish peroxidase (HRP) was supplemented. Finally, development was completed in line with the electro‐chemiluminescence detection kit (Vazyme Biotech, Nanjing, China), and the greyscales were analysed on the gel imager (model: 2000; Alpha, San Antonio, TX, USA).

### 2.11 Dual luciferase reporter gene assay

We predicted the potential binding sites of XIST and miR‐30a‐5p by adoption of Starbase software (http://starbase.sysu.edu.cn/ago-ClipRNA.php?source=lncRNA&flag=target&clade=mammal&genome=human&assembly=hg19&miRNA=all&clipNum=1&deNum=0&panNum=0&target=XIST).^18^ The XIST and ROR1 fragments that incorporated binding sites with miR‐30a‐5p were amplified through performing PCR at first and were then inserted into pmirGLO (Promega, Madison, WI, USA) in order to establish the reporter vectors of pmirGLO‐XIST Wt and pmirGLO‐ROR1 Wt. For another, the reporter vectors of pmirGLO‐XIST Mut and pmirGLO‐ROR1 Mut were constructed through mutating the binding sites of XIST and ROR1 to miR‐30a‐5p within XIST and ROR1 fragments. With the help of Lipofectamine^™^2000 transfection kit (Promega, Madison, WI, USA), pmirGLO‐XIST Mut, pmirGLO‐XIST Wt, pmirGLO‐ROR1 Mut and pmirGLO‐ROR1 Wt were, respectively, co‐transfected with miR‐30a‐5p mimic or miR‐NC into CRC cells. After 48‐h transfection, the luciferase reporter gene activity of cells was determined following the guidance of dual luciferase detection kit (Promega, Madison, WI, USA). The activation degree of samples was obtained through dividing the relative light unit (RLU) of firefly luciferase by the RLU of Renilla luciferase.

### 2.12 Statistical analyses

All the statistical analyses were implemented by utilizing SPSS17.0 software (Version X; IBM, Armonk, NY, USA). The enumeration data in the form of mean ± standard deviation (SD) were analysed through Student's *t* test for between‐group comparisons or via one‐way analysis of variance (ANOVA) with Bonferroni test for among‐group comparisons. The correlations between genetic expressions and the clinicopathological features of CRC patients were evaluated by way of Spearman correlation test, and the survival analysis was accomplished by carrying out Kaplan‐Meier analysis. It would be considered statistically significant when *P *<* *0.05.

## 3 RESULTS

### 3.1 Association of XIST and miR‐30a‐5p expressions with clinicopathological features of CRC patients

**Figure 1:**
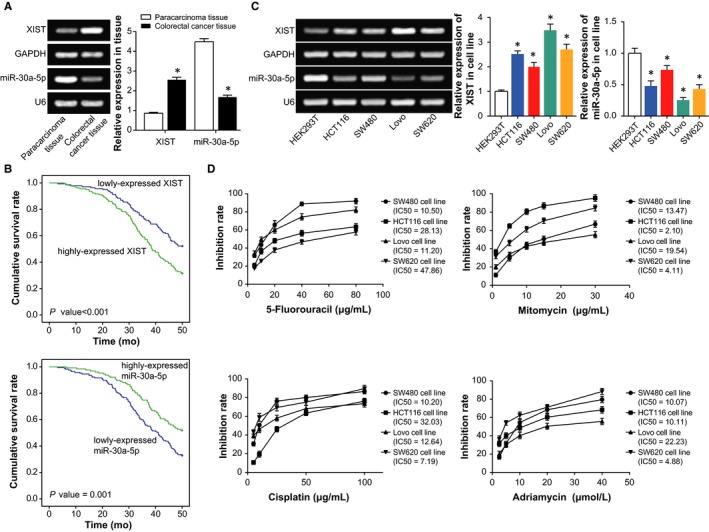
The association of XIST and miR‐30a‐5p expressions with onset and prognosis of colorectal cancer patients, and colorectal cancer cells were selected regarding their resistance to chemotherapies. A, XIST and miR‐30a‐5p expressions were compared between colorectal cancer tissues and paracarcinoma tissues. *P <* *0.05 when compared with paracarcinoma tissues. B, Expressions of XIST and miR‐30a‐5p were compared among HEK293T, SW480, HCT116, Lovo and SW620 cell lines. *P <* *0.05 when compared with HEK293T. C, Lowly expressed XIST and highly expressed miR‐30a‐5p were correlated with more favourable overall survival of colorectal cancer patients than highly expressed XIST and lowly expressed miR‐30a‐5p respectively. D, The sensitivities of SW480, HCT116, Lovo and SW620 cell lines were compared in response to 5‐fluorouracil, mitomycin, cisplatin and adriamycin

As illustrated in Figure 1A, XIST expression within CRC tissues was around 2.98 folds of that within paracarcinoma normal tissues (*P *<* *0.05), while expression of miR‐30a‐5p within CRC tissues achieved only 37% of that within the normal tissues (*P *<* *0.05). Moreover, the included CRC patients were grouped into ones with highly expressed XIST (>2.56) and ones with lowly expressed XIST (≤2.56) based on the median level of XIST expression. The same population was also categorized into highly expressed miR‐30a‐5p group (>1.68) and ones with lowly expressed miR‐30a‐5p group (≤1.68) in line with the median level of miR‐30a‐5p expression. It was exhibited that overexpressed XIST and under‐expressed miR‐30a‐5p were more frequently detected in CRC patients characterized by large tumours (4.255, *P *=* *0.039; 5.173, *P *=* *0.023), advanced clinical stage (7.373, *P *=* *0.007; 6.264, *P *=* *0.012) and lymphatic metastasis (5.872, *P *=* *0.015; 4.364, *P *=* *0.037), when compared with under‐expressed XIST and overexpressed miR‐30a‐5p (Table 2). Besides, the CRC subjects with overexpressed XIST and under‐expressed miR‐30a‐5p, respectively, displayed poorer prognosis than under‐expressed XIST (HR* *=* *2.26, 95% CI: 1.32‐3.88, *P *=* *0.003) and overexpressed miR‐30a‐5p (HR* *=* *1.97, 95% CI: 1.15‐3.37, *P *=* *0.013) (Figure 1B) (Table 3).

### 3.2 Comparison of chemo‐resistance among CRC cell lines

With HEK293T cell line as the control, markedly raised XIST expression and lowered miR‐30a‐5p expression were determined within SW480, Lovo, HCT116 and SW620 cell lines (*P *<* *0.05) (Figure 1C). Interestingly, the highly metastatic Lovo cell line showed the topmost XIST expression and the minimum miR‐30a‐5p expression (*P *<* *0.05), yet the non‐metastatic and tumour‐generating SW480 cell line was correlated with the highest miR‐30a‐5p expression and yet the lowest XIST expression among the CRC cell lines studied (*P *<* *0.05).

Furthermore, Lovo cell line presented stronger resistances to mitomycin (IC50 = 19.54 μg/mL) and adriamycin (IC50 = 22.23 μmol/L) than any other cell line (*P *<* *0.05). Besides, under treatment of cisplatin, HCT116 cell line (IC50 = 32.03 μg/mL) and Lovo cell line (IC50 12.64 μg/mL), respectively, exhibited the highest and the second highest resistances. As for 5‐fluorouracil, the resistance of cells was ranked as: SW620 (IC50 = 47.86 μg/mL) > HCT116 (IC50 = 28.13 μg/mL) > Lovo (IC50 = 11.20 μg/mL) > 5‐Fu (IC50 = 10.50 μg/mL) (Figure 1D). Considering that Lovo cell line and SW480 cell line, respectively, exhibited higher and lower resistance to the four drugs than any other cells, they were managed for the following experiments.

### 3.3 Regulatory contribution of XIST and miR‐30a‐5p to chemosensitivity of CRC cells

**Figure 2:**
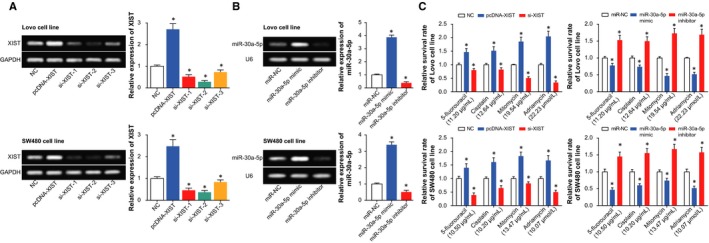
The impacts of XIST and miR‐30a‐5p on the response of colorectal cancer cells to drugs. A, XIST expression was determined after transfection of pcDNA‐XIST or si‐XIST. *P < 0.05 when compared with NC. B, The expression of miR‐30a‐5p was measured after transfection of miR‐30a‐5p mimic or miR‐30a‐5p inhibitor. *P <* *0.05 when compared with NC. C, The sensitivity of colorectal cells to 5‐fluorouracil, mitomycin, cisplatin and adriamycin was compared when XIST and miR‐30a‐5p expressions were up‐regulated and down‐regulated. *P <* *0.05 when compared with NC

Among the 3 si‐XISTs adopted, it was indicated that si‐XIST‐3 presented a far stronger capacity to inhibit XIST expression than si‐XIST‐1 and si‐XIST‐2 (*P *<* *0.05), so si‐XIST‐3 was prepared for the following experiments (Figure 2A). After transfection of pcDNA‐XIST or si‐XIST3, the expression of XIST was, respectively, brought up and down with statistical significance (*P *<* *0.05) (Figure 2A). Conversely, miR‐30a‐5p expression was markedly raised and reduced, respectively, under transfections of miR‐30a‐5p mimic and miR‐30a‐5p inhibitor (*P *<* *0.05) (Figure 2B). Against the contexts of promoted XIST expression or restrained miR‐30a‐5p expression, the Lovo and SW480 cell line took on enhancive survival in response to treatments of 5‐fluorouracil, mitomycin, adriamycin and cisplatin at their IC50 concentrations for each cell line (*P *<* *0.05) (Figure 2C). Nonetheless, transfection of si‐XIST2 or miR‐30a‐5p mimic hindered the survival rate of Lovo and SW480 cell line, when compared with NC group (*P *<* *0.05).

### 3.4 Impacts of XIST and miR‐30a‐5p on the viability, proliferation and apoptosis of CRC cells

**Figure 3:**
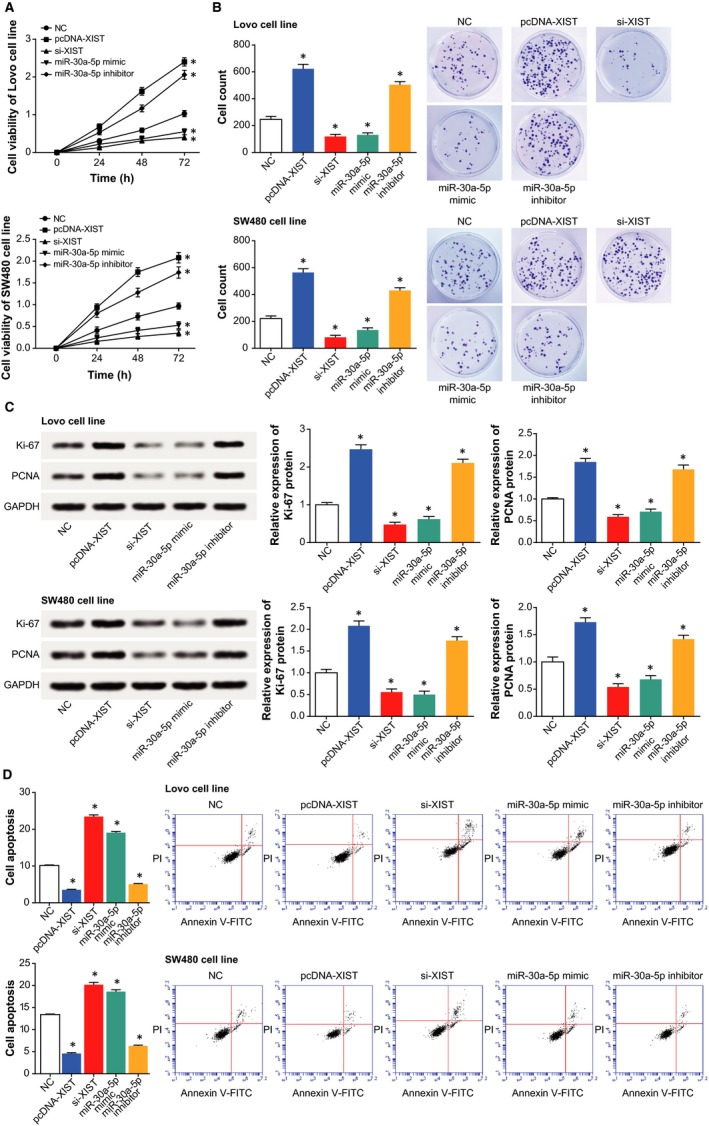
The influences of XIST and miR‐30a‐5p on viability, proliferation and apoptosis of colorectal cancer cells. A, The viabilities of colorectal cancer cells were determined after respective transfections of pcDNA‐XIST, si‐XIST, miR‐30a‐5p mimic and miR‐30a‐5p inhibitor. *P <* *0.05 when compared with NC. B, The proliferative capacities of colorectal cancer cells were compared among cells transfected with pcDNA‐XIST, si‐XIST, miR‐30a‐5p mimic and miR‐30a‐5p inhibitor. *P <* *0.05 when compared with NC. C, The expressions of cell growth factors (ie Ki‐67 and PCNA) were compared after transfection of pcDNA‐XIST, si‐XIST, miR‐30a‐5p mimic and miR‐30a‐5p inhibitor. *P <* *0.05 when compared with NC. D, The apoptotic percentages of cells were assessed under the influences of pcDNA‐XIST, si‐XIST, miR‐30a‐5p mimic and miR‐30a‐5p inhibitor. *P <* *0.05 when compared with NC

Under conditions of under‐expressed XIST or overexpressed miR‐30a‐5p, we observed that the viability and proliferation of cells were significantly prohibited (*P *<* *0.05) (Figure 3A,B), yet cell apoptosis was improved (*P *<* *0.05) (Figure 3D). Nevertheless, cells treated with pcDNA‐XIST and miR‐30a‐5p inhibitor were linked with encouraged viability and proliferation (*P *<* *0.05), along with depressed apoptosis (*P *<* *0.05). Furthermore, addition of pcDNA‐XIST and miR‐30a‐5p inhibitor greatly up‐regulated biomarkers relevant to cell proliferation (ie Ki‐67 and PCNA), yet si‐XIST2 and miR‐30a‐5p mimic motivated an opposite trend (*P *<* *0.05) (Figure 3C).

### 3.5 MiR‐30a‐5p was subjected to modulation of XIST

**Figure 4:**
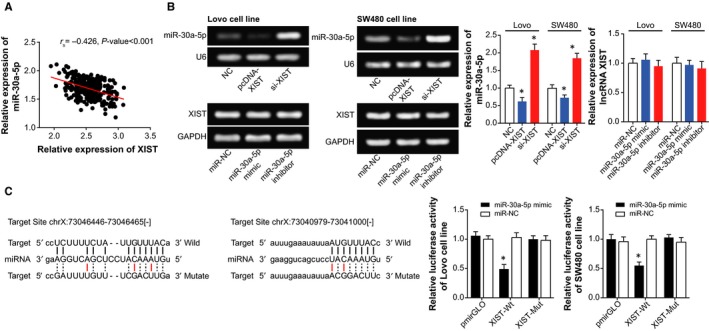
The relationship between XIST and miR‐30a‐5p in colorectal cancer cells. A, The XIST expression was negatively correlated with miR‐30a‐5p expression among the included colorectal cancer tissues. B, MiR‐30a‐5p expression was determined after respective transfection of pcDNA‐XIST and si‐XIST. *P <* *0.05 when compared with NC. C, XIST targeted miR‐30a‐5p in certain sites, and the luciferase activities of cells were compared among pmirGLO‐XIST‐Wt+miR‐30a‐5p mimic, pmirGLO‐XIST‐Mut+miR‐30a‐5p mimic, and pmirGLO+miiR‐30a‐5p mimic groups. *P <* *0.05 when compared with pmirGLO+miR‐30a‐5p mimic group

Among the incorporated CRC tissues, XIST expression was negatively correlated with miR‐30a‐5p expression (*r*_s_ **=* *−0.426, *P *<* *0.001) (Figure 4A). Moreover, transfection of pcDNA‐XIST and si‐XIST2 could both alter the expression of miR‐30a‐5p significantly (*P *<* *0.05); however, transfection of either miR‐30a‐5p mimic or miR‐30a‐5p inhibitor hardly changed the expression of XIST (*P *<* *0.05) (Figure 4B). With regard to dual luciferase reporter gene assay, the luciferase activity of pmirGLO‐XIST‐Wt+miR‐30a‐5p mimic group was decreased in comparison to pmirGLO‐XIST‐Mut+miR‐30a‐5p mimic group (*P *<* *0.05) (Figure 4C). Nevertheless, after mutation of bases within the binding site of XIST and miRNA‐30a‐5p, over‐expression of miRNA‐30a‐5p would no longer affect the fluorescent activity of luciferase plasmids (*P *>* *0.05).

### 3.6 ROR1 mediated the role of miR‐30a‐5p in regulating chemosensitivity of CRC cells

**Figure 5:**
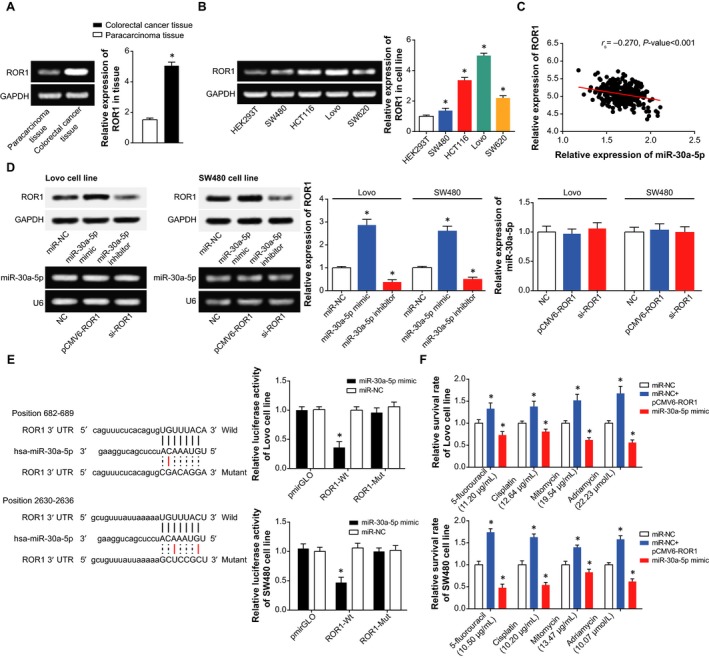
The mediation of ROR1 for the contributions of XIST and miR‐30a‐5p to chemosensitivity of colorectal cancer cells. A, The expression of ROR1 was compared between colorectal cancer tissues and paracarcinoma tissues. *P <* *0.05 when compared with paracarcinoma tissues. B, The expression of ROR1 was determined within HEK293T, SW480, HCT116, Lovo and SW620 cell lines. *P <* *0.05 when compared with HEK293T. C, Among the incorporated colorectal cancer tissues, ROR1 expression was positively correlated with XIST expression, yet it displayed negative relevance to miR‐30a‐5p. D, The expression of ROR1 was detected after transfections of pcDNA‐XIST, s‐XIST, miR‐30a‐5p mimic and miR‐30a‐5p inhibitor, and the expressions of XIST and miR‐30a‐5p were also determined after transfections of pCMV1‐ROR1 and si‐ROR1. *P <* *0.05 when compared with NC. E, ROR1 was subjected to target of miR‐30a‐5p in certain sites, and the luciferase activity of cells was compared among miR‐30a‐5p mimic+pmirGLO‐ROR1‐Wt, miR‐30a‐5p mimic+pmirGLO‐ROR1‐Mut and miR‐30a‐5p mimic+pmirGLO groups. *P <* *0.05 when compared with pmirGLO+miR‐30a‐5p mimic group. F, The sensitivity of colorectal cancer cells was compared when responding to 5‐fluorouracil, mitomycin, cisplatin and adriamycin among the miRNA‐NC, miR‐30a‐5p mimic and miR‐NC+pCMV1‐ROR1 groups. *P <* *0.05 when compared with NC

It was indicated in Figure 5A,B that ROR1 expression within human CRC tissues and cell lines (ie SW480, HCT116, Lovo and SW620) surpassed that within paracarcinoma tissues and HEK293T cell line (*P *<* *0.05). What was more, ROR1 expression was negatively correlated with miR‐30a‐5p expression among the CRC tissues included (*r*_s_ **=* *−0.270, *P *<* *0.001) (Figure 5C). Also transfection of miR‐30a‐5p mimic and inhibitor, respectively, down‐regulated and up‐regulated ROR1 expression (*P *<* *0.05), yet ROR1 exerted no effects on the expression of miR‐30a‐5p (*P *>* *0.05) (Figure 5D). As for the relationship between miR‐30a‐5p and ROR1, the wide‐type ROR1 and miR‐30a‐5p mimic could reduce the luciferase activity of plasmids (*P *<* *0.05), but the mutated ROR1 failed to influence the luciferase activity (*P *>* *0.05) (Figure 5E). To sum up, it was implied that ROR1 was modified by miR‐30a‐5p in CRC.

**Figure 6:**
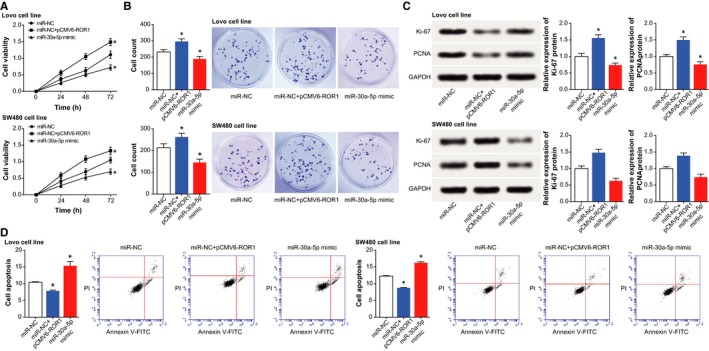
The role of ROR1 in mediating the modulatory impacts of miR‐30a‐5p on the activities of colorectal cancer cells. A, The viabilities of colorectal cancer cells were compared among the miRNA‐NC, miR‐30a‐5p mimic and miR‐NC+pCMV1‐ROR1 groups. *P <* *0.05 when compared with NC. B, The proliferation of colorectal cancer cells were compared among cells transfected with miRNA‐NC, miR‐30a‐5p mimic and miR‐NC+pCMV1‐ROR1. *P <* *0.05 when compared with NC. C, The expressions of cell growth factors (ie Ki‐67 and PCNA) were evaluated after transfections of miRNA‐NC, miR‐30a‐5p mimic and miR‐NC+pCMV1‐ROR1. *P <* *0.05 when compared with NC. D, The apoptotic percentages of cells were assessed under the influences of miRNA‐NC, miR‐30a‐5p mimic and miR‐NC+pCMV1‐ROR1. *P <* *0.05 when compared with NC

In addition, miR‐NC+pCMV1‐ROR1 group appeared to boost the resistance of Lovo cell line to drugs when compared with miR‐30a‐5p mimic group (*P *<* *0.05), though its contribution to chemosensitivity was below that of miR‐NC group (*P *<* *0.05) (Figure 5F). Meanwhile, we observed that the viability and proliferation of CRC cells were encouraged the apoptotic condition of the cells was suppressed after co‐transfection of miR‐NC and pCMV1‐ROR1, with miR‐30a‐5p mimic group as the reference (*P *<* *0.05) (Figure 6). Correspondingly, miR‐NC+pcDNA‐ROR1 group potently prohibited apoptosis of CRC cells and improved their viability and proliferation more significantly than miR‐30a‐5p mimic group and miR‐NC group (*P *<* *0.05).

### 3.7 AO‐II promoted chemosensitivity of CRC cells by modifying XIST/miR‐30a‐3p/ROR 1 axis

**Figure 7:**
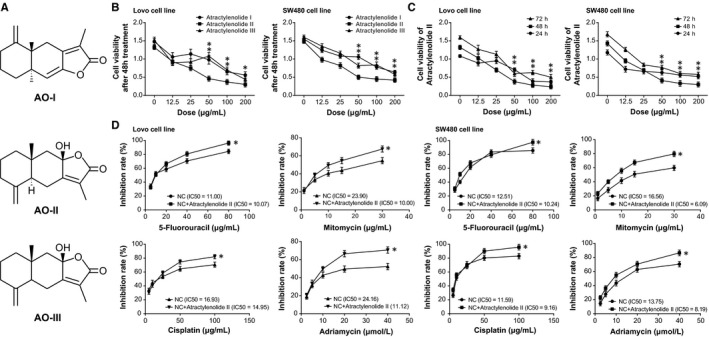
The atractylenolide I, atractylenolide II and atractylenolide III were compared regarding their sensitivity to chemo drugs. A, The structure drawing of atractylenolide I, atractylenolide II and atractylenolide III. B, The atractylenolide I, atractylenolide II and atractylenolide III at the concentrations of 0, 12.5, 25, 50, 100 and 200 μg/mL were assessed regarding their impacts on the viability of colorectal cancer cells. *P <* *0.05 when compared with atractylenolide II. C, The atractylenolide II at the concentrations of 0, 12.5, 25, 50, 100 and 200 μg/mL was assessed concerning its contributions to the viability of colorectal cancer cells at the time‐points of post‐treatment 24, 48 and 72 h. *P <* *0.05 when compared with the 48 h. D, The atractylenolide II was compared regarding its acting on the sensitivity of colorectal cancer cells in response to 5‐fluorouracil, mitomycin, cisplatin and adriamycin were compared. *P <* *0.05 when compared with NC

After treating CRC cells with AO‐I, AO‐II and AO‐III (Figure 7A) for 48 hours, it was discovered that AO‐I, AO‐II and AO‐III at the dosage of 50 μg/mL all showed obvious inhibition to proliferation of the cells (*P *<* *0.05) (Figure 7B). Moreover, AO‐II at the concentration of 50 μg/mL produced greater inhibition than AO‐I and AO‐III at the same dosage (*P *<* *0.05). According to the above results, AO‐II at different dosages was selected for conduction of time‐effect experiments as Figure 7C. It was manifested that the suppressive impact of AO‐II at the dosage of 50 μg/mL peaked at 48 hours after administration. Besides, addition of AO‐II significantly elevated the sensitivity of CRC cells to 5‐fluorouracil (IC50 = 10.07 μg/mL vs 10.24 μg/mL), mitomycin (IC50 = 10.00 μg/mL vs 6.09 μg/mL), adriamycin (IC50 = 11.12 μmol/L vs 8.19 μmol/L) and cisplatin (IC50 = 14.95 μg/mL vs 9.16 μg/mL) (Figure 7D) (*P *<* *0.05).

**Figure 8:**
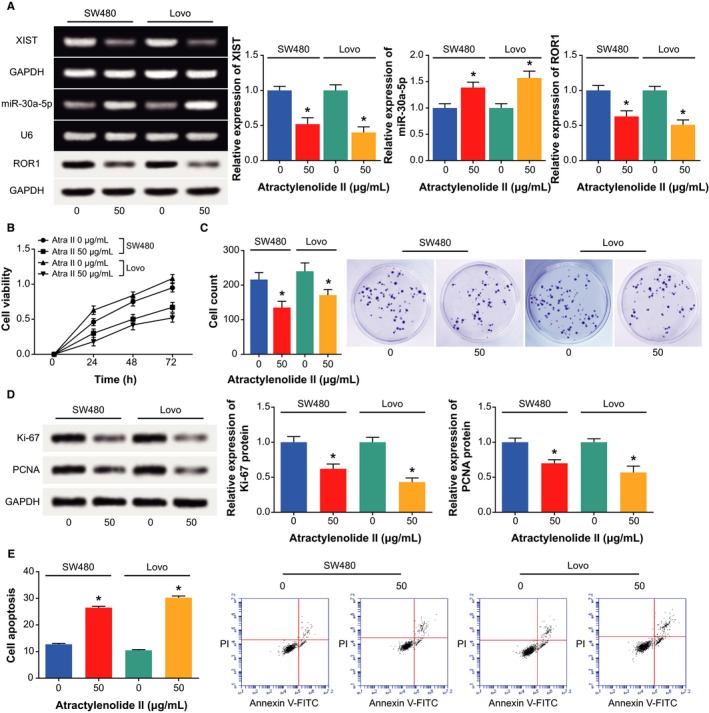
The reversal function of atractylenolide II for chemosensitivity of colorectal cancer cells via modifying XIST/miR‐30a‐3p/ROR 1 axis. A, The expressions of XIST, miR‐30a‐3p and ROR 1 within colorectal cancer cells were determined after additions of atractylenolide II. *P <* *0.05 when compared with NC. B, The viability of colorectal cancer cells was evaluated after supplementation of atractylenolide II. *P <* *0.05 when compared with NC. C, The proliferative capability of colorectal cancer cells was appraised after being treated with atractylenolide II. *P <* *0.05 when compared with NC. D, The expressions of ki‐67 and PCNA were determined within colorectal cancer cells under the influence of atractylenolide II. *P <* *0.05 when compared with NC. E, The apoptosis of colorectal cancer cells was evaluated under the action of atractylenolide II. *P <* *0.05 when compared with NC

As shown in Figure 8A, 48‐hour treatment of CRC cells with 50 μg/mL AO‐II could bring down the expressions of XIST and ROR1, when compared with NC group (*P *<* *0.05). Distinct from XIST and ROR1, the expression of miR‐30a‐3p expression was intensified (*P *<* *0.05). Simultaneously, after treatment of AO‐II, the viability of CRC cells was impaired, and the proliferative capacity of the cells was hindered, in comparison to NC group (*P *<* *0.05) (Figure 8B‐D). By contrast, AO‐II generated higher apoptotic percentages than NC group (*P *<* *0.05) (Figure 8E).

## 4 DISCUSSION

The main approaches for treating CRC covered surgical treatment, chemotherapy, radiotherapy and TCM treatment, among which chemotherapy, as a valid systemic therapy, was especially beneficial to patients who did not meet the surgical indications. Although novel anticancer drugs and combined chemotherapies have sprung up, the chemotherapeutic efficacy for CRC was reinforced indistinctively, owing to the gradual production of drug resistance and accompanying toxicity. The primary drug resistance and multidrug resistance appeared as the tough obstacle to tumour chemotherapy, therefore, reversing the drug resistance of tumour cells has become the key to improve treatment efficacy for neoplasms.

Incremental evidence indicated that the XIST investigated here was tightly linked with occurrence and progression of tumours, and it counted in maintaining the normal state of genome.^16^, ^36^ For example, highly expressed XIST was present concurrently with larger tumour volume, metastatic lymph node, distant metastasis and advanced TNM staging of patients with gastric cancer.^37^, ^38^ Furthermore, knockout of XIST exhibited a role in inhibiting progression of glioblastoma by reducing cell proliferation, migration and invasion, as well as inducing cell apoptosis.^15^ Analogously, XIST expression was up‐regulated within CRC tissues, as was illustrated in Figure 1. In addition, it was reported that XIST expression was negatively correlated with the median lethal concentration of paclitaxel in treating ovarian cancer cells, suggesting that cell lines with down‐regulated XIST expression exhibited descending sensitivity to treatment of paclitaxel.^39^ The XIST also displayed up‐regulated expression within CRC cells that presented resistance to 5‐fluorouracil.^40^ Within our investigation, the function of XIST in CRC was expanded that XIST could favour resistance of CRC cells to 5‐fluorouracil, mitomycin, adriamycin and cisplatin (Figure 2), and this phenomenon was conjectured to the result of XIST’ facilitating proliferation and viability of CRC cells (Figure 3).

Besides, a targeted relationship between XIST and miR‐30a was built here, and XIST was identified to down‐regulate miR‐30a expression in CRC through targeting it (Figure 4). Virtually, apart from CRC,^41^ the miR‐30a, located in the intron region of human chromosome 6q13, has also been suggested as a protector against progression of liver cancer,^42^, ^43^ renal clear cell carcinoma,^44^ gastric cancer,^45^ prostate cancer^46^ and breast cancer.^47^ The tumour‐suppressing potential of miR‐30a was speculated as the result of its obstructing epithelial‐mesenchymal transition and metastasis of lung cancer, hepatoma carcinoma and breast cancer cells.^21^, ^42^, ^48^ Consistent with the malignancies, our results also indicated miR‐30a as a marker promoting the prognosis of CRC patients (Figure 1), and the intrinsic pathology was specifically embodied in its restraining viability and proliferation of CRC cells (Figure 3). Based on the experimental evidence and reasoning, it was convincing that XIST collaborating with miR‐30a could modify the chemo‐resistance of CRC cells (Tables 2 and 3).

**Table 2:** Correlation between XIST and miR‐30a‐5p expressions and baseline characteristics of colorectal cancer patients

Characteristics	LncRNA XIST expression				miR‐30a‐5p expression			
	Lower	Higher	χ^2^	*P* value	Lower	Higher	χ^2^	*P* value
Age (years)								
>60	57	80	2.929	0.087	70	67	3.406	0.065
≤60	81	76			97	60		
Gender								
Male	77	83	0.198	0.656	90	70	0.044	0.834
Female	61	73			77	57		
Tumour size (cm)								
>5	44	68	**4.255**	**0.039**	73	39	**5.173**	**0.023**
≤5	94	88			94	88		
Location								
Colon	78	75	2.092	0.148	89	64	0.243	0.622
Rectum	60	81			78	63		
Differentiation								
Poorly	18	34	3.852	0.050	34	18	1.896	0.169
Well and moderately	120	122			133	109		
Depth of tumour								
T3 + T4	101	134	**7.373**	**0.007**	142	93	**6.264**	**0.012**
T1 + T2	37	22			25	34		
Lymphatic invasion								
Presence	71	102	**5.872**	**0.015**	107	66	**4.364**	**0.037**
Absence	67	54			60	61		
Distant metastasis								
Presence	15	24	1.297	0.255	20	19	0.559	0.455
Absence	123	132			147	108		
TNM stage								
III + IV	74	99	2.927	0.087	105	68	2.594	0.107
I + II	64	57			62	59		

The bold value indicate a significant results with a *P* < 0.05.

**Table 3:** Correlation between clinical characteristics of significance and overall survival of colorectal cancer patients

Characteristics	Univariate analysis			Multivariate analysis		
	Hazard ratio	95% CI	*P* value	Hazard ratio	95% CI	*P* value
XIST expression						
High vs Low	2.38	1.48‐3.83	**<0.001**	2.26	1.32‐3.88	**0.003**
miR‐30a‐5p expression						
Low vs High	2.20	1.37‐3.54	**0.001**	1.97	1.15‐3.37	**0.013**
Age (years)						
>60 vs ≤60	0.73	0.46‐1.16	0.183	0.61	0.36‐1.04	0.071
Gender						
Male vs Female	0.99	0.62‐1.58	0.972	1.05	0.62‐1.77	0.855
Tumour size (cm)						
>5 vs ≤5	2.93	1.75‐4.89	**<0.001**	2.67	1.53‐4.65	**0.001**
Location						
Colon vs Rectum	0.80	0.50‐1.27	0.339	0.87	0.52‐1.46	0.598
Differentiation						
Poorly vs Well and moderately	1.55	0.82‐2.92	0.174	1.32	0.65‐2.66	0.441
Depth of tumour						
T3 + T4 vs T1 + T2	2.31	1.30‐4.13	**0.005**	1.90	0.99‐3.63	0.051
Lymphatic invasion						
Presence vs Absence	2.15	1.34‐3.47	**0.002**	1.71	1.02‐2.88	**0.043**
Distant metastasis						
Presence vs Absence	1.01	0.51‐2.00	0.986	1.02	0.48‐2.18	0.950
TNM stage						
III + IV vs I + II	1.28	0.80‐2.04	0.312	1.03	0.61‐1.75	0.900

The bold value indicate a significant results with a *P* < 0.05.

In addition, this investigation ascertained that miR‐30a‐5p could down‐regulate ROR1 expression to suppress the invasive and migratory potency of CRC cells (Figure 5). And ROR1 was also testified as the downstream mediator of miR‐30a‐5p and XIST in modulating chemosensitivity of CRC cells (Figure 6). The ROR1 studied herein was a membrane‐spanning protein belonging to the receptor tyrosine kinase (RTK) family, and it was aberrantly overexpressed within multiple haematological and solid malignancies, such as lymphatic leukaemia, melanoma and ovarian cancer.^49^, ^50^, ^51^, ^52^, ^53^ In terms of molecular mechanisms, furthermore, the ROR1 was inclined to activate signal transducer and activator of transcription 3 (STAT3), phosphatidylinositol 3‐kinase (PI3K) and cellular‐mesenchymal to epithelial transition factor (c‐Met), which appeared essential to modification of tumour growth and metastasis.^54^, ^55^, ^56^ In fact, the involvement of ROR1 in neoplasms was also subjected to the control of other miRNAs, such as miR‐382 in ovarian cancer and miR‐30a in breast cancer.^25^, ^57^ It was insinuated that a molecular network among miRNAs and genes was existent underlying the aetiology of CRC, and the contribution of ROR1 to CRC progression could also be modulated by several other upstream miRNAs, which demanded in‐depth researches.

Concerning the TCM drug, we observed that the AOs could attenuate the chemosensitivity of CRC cells through weakening viability and proliferation of CRC cells (Figure 7). The results followed the same principle with previous documentations, which believed that AO‐I was adept at inhibiting growth of human white blood cell strain and mouse leukaemia cell lines.^34^ Moreover, AO‐II could serve to control the proliferation of melanoma cells,^58^ and AO‐III triggered depressed tumour growth and incremental release of lactic dehydrogenase.^59^ The above proof all pointed to a direction that AO tended to postpone neoplastic deterioration, which suggested a linkage of AO with elevated chemosensitivity of cancer cells. We found that AO‐II was equipped with the strongest ability to inhibit chemo‐resistance of CRC cells among the 3 AOs, and addition of them was associated with down‐regulated XIST and ROR1 expressions, along with up‐regulated miR‐30a‐5p expression (Figure 8). It was thus insinuated that the AO‐II might work for incremental chemo‐resistance of CRC cells by fighting against the contribution of XIST/miR‐30a‐5p/ROR1 axis to CRC development. As for the additional particularized mechanism related with AO‐II and CRC, extra investigations were demanded.

In conclusion, this investigation demonstrated the vital role of LncRNA XIST/miR‐30a‐5p/ROR1 axis in altering chemosensitivity of CRC cells, and AO‐II also played a part contrary to the signalling axis. The conclusion of this investigation might help to ameliorate the chemotherapeutic efficacy for CRC.

## CONFLICT OF INTEREST

None.

## References

[1] Aoyagi T , Terracina KP , Raza A , Takabe K . Current treatment options for colon cancer peritoneal carcinomatosis. World J Gastroenterol. 2014;20:12493‐12500. 25253949 10.3748/wjg.v20.i35.12493 PMC4168082

[2] Wang RJ , Wu P , Cai GX , et al. Down‐regulated MYH11 expression correlates with poor prognosis in stage II and III colorectal cancer. Asian Pac J Cancer Prev. 2014;15:7223‐7228. 25227818 10.7314/apjcp.2014.15.17.7223

[3] Douillard JY , Oliner KS , Siena S , et al. Panitumumab‐FOLFOX4 treatment and RAS mutations in colorectal cancer. N Engl J Med. 2013;369:1023‐1034. 24024839 10.1056/NEJMoa1305275

[4] O'Connell JB , Maggard MA , Ko CY . Colon cancer survival rates with the new American Joint Committee on Cancer sixth edition staging. J Natl Cancer Inst. 2004;96:1420‐1425. 15467030 10.1093/jnci/djh275

[5] Alaiyan B , Ilyayev N , Stojadinovic A , et al. Differential expression of colon cancer associated transcript1 (CCAT1) along the colonic adenoma‐carcinoma sequence. BMC Cancer. 2013;13:196. 23594791 10.1186/1471-2407-13-196 PMC3639026

[6] Han P , Li JW , Zhang BM , et al. The lncRNA CRNDE promotes colorectal cancer cell proliferation and chemoresistance via miR‐181a‐5p‐mediated regulation of Wnt/beta‐catenin signaling. Mol Cancer. 2017;16:9. 28086904 10.1186/s12943-017-0583-1 PMC5237133

[7] Yin DD , Liu ZJ , Zhang E , Kong R , Zhang ZH , Guo RH . Decreased expression of long noncoding RNA MEG3 affects cell proliferation and predicts a poor prognosis in patients with colorectal cancer. Tumour Biol. 2015;36:4851‐4859. 25636452 10.1007/s13277-015-3139-2

[8] Xu C , Yang M , Tian J , Wang X , Li Z . MALAT‐1: a long non‐coding RNA and its important 3’ end functional motif in colorectal cancer metastasis. Int J Oncol. 2011;39:169‐175. 21503572 10.3892/ijo.2011.1007

[9] Shi D , Zheng H , Zhuo C , et al. Low expression of novel lncRNA RP11‐462C24.1 suggests a biomarker of poor prognosis in colorectal cancer. Med Oncol. 2014;31:31. 24908062 10.1007/s12032-014-0031-7 PMC4079943

[10] Song H , He P , Shao T , Li Y , Li J , Zhang Y . Long non‐coding RNA XIST functions as an oncogene in human colorectal cancer by targeting miR‐132‐3p. J BUON. 2017;22:696‐703. 28730777

[11] Dou J , Ni Y , He X , et al. Decreasing lncRNA HOTAIR expression inhibits human colorectal cancer stem cells. Am J Transl Res. 2016;8:98‐108. 27069543 PMC4759419

[12] Brown CJ , Ballabio A , Rupert JL , et al. A gene from the region of the human X inactivation centre is expressed exclusively from the inactive X chromosome. Nature. 1991;349:38‐44. 1985261 10.1038/349038a0

[13] Weakley SM , Wang H , Yao Q , Chen C . Expression and function of a large non‐coding RNA gene XIST in human cancer. World J Surg. 2011;35:1751‐1756. 21212949 10.1007/s00268-010-0951-0 PMC3275083

[14] Yildirim E , Kirby JE , Brown DE , et al. Xist RNA is a potent suppressor of hematologic cancer in mice. Cell. 2013;152:727‐742. 23415223 10.1016/j.cell.2013.01.034 PMC3875356

[15] Nogami M . Reduction mechanism for Eu ions in Al2O3‐containing glasses by heat treatment in H2 gas. J Phys Chem B. 2015;119:1778‐1784. 25574780 10.1021/jp511513n

[16] Tantai J , Hu D , Yang Y , Geng J . Combined identification of long non‐coding RNA XIST and HIF1A‐AS1 in serum as an effective screening for non‐small cell lung cancer. Int J Clin Exp Pathol. 2015;8:7887‐7895. 26339353 PMC4555681

[17] Zhang J , Zhang H , Xu X , Wang M , Yu Z . Comparative genomic hybridization analysis of invasive ductal breast carcinomas in the Chinese population. Oncol Lett. 2015;10:2100‐2106. 26622803 10.3892/ol.2015.3608 PMC4579858

[18] Li JH , Liu S , Zhou H , Qu LH , Yang JH . starBase v2.0: decoding miRNA‐ceRNA, miRNA‐ncRNA and protein‐RNA interaction networks from large‐scale CLIP‐Seq data. Nucleic Acids Res. 2014;42:D92‐D97. 24297251 10.1093/nar/gkt1248 PMC3964941

[19] Zeng RC , Zhang W , Yan XQ , et al. Down‐regulation of miRNA‐30a in human plasma is a novel marker for breast cancer. Med Oncol. 2013;30:477. 23389917 10.1007/s12032-013-0477-z

[20] Zheng B , Zhu H , Gu D , et al. MiRNA‐30a‐mediated autophagy inhibition sensitizes renal cell carcinoma cells to sorafenib. Biochem Biophys Res Commun. 2015;459:234‐239. 25712526 10.1016/j.bbrc.2015.02.084

[21] Kumarswamy R , Mudduluru G , Ceppi P , et al. MicroRNA‐30a inhibits epithelial‐to‐mesenchymal transition by targeting Snai1 and is downregulated in non‐small cell lung cancer. Int J Cancer. 2012;130:2044‐2053. 21633953 10.1002/ijc.26218

[22] Mazeh H , Mizrahi I , Ilyayev N , et al. The diagnostic and prognostic role of microRNA in colorectal cancer – a comprehensive review. J Cancer. 2013;4:281‐295. 23459799 10.7150/jca.5836 PMC3584841

[23] Wang P , Liang J , Li Y , et al. Down‐regulation of miRNA‐30a alleviates cerebral ischemic injury through enhancing beclin 1‐mediated autophagy. Neurochem Res. 2014;39:1279‐1291. 24771295 10.1007/s11064-014-1310-6

[24] Zhu H , Wu H , Liu X , et al. Regulation of autophagy by a beclin 1‐targeted microRNA, miR‐30a, in cancer cells. Autophagy. 2009;5:816‐823. 19535919 10.4161/auto.9064 PMC3669137

[25] Wang X , Qiu H , Tang R , et al. miR30a inhibits epithelialmesenchymal transition and metastasis in triplenegative breast cancer by targeting ROR1. Oncol Rep. 2018;39:2635‐2643. 29693179 10.3892/or.2018.6379 PMC5983935

[26] Zhou JK , Zheng YZ , Liu XS , et al. ROR1 expression as a biomarker for predicting prognosis in patients with colorectal cancer. Oncotarget. 2017;8:32864‐32872. 28427197 10.18632/oncotarget.15860 PMC5464834

[27] Yang X , Bai F , Xu Y , Chen Y , Chen L . Intensified beclin‐1 mediated by low expression of Mir‐30a‐5p promotes chemoresistance in human small cell lung cancer. Cell Physiol Biochem. 2017;43:1126‐1139. 28977798 10.1159/000481754

[28] Li G , Zhao Y . Treatment of diarrhea after radiotherapy and chemotherapy with Shenling Baizhu San. Clin Med. 2006;26:85‐86.

[29] Zhang C , Zhang Y , Jiang D , Han S . Clinical study on the effect of TCM yiqi jianpi method on leukocyte growth after chemotherapy in patients with ovarian cancer. Community Health Care. 2004;2:118‐121.

[30] Guan X , Qu X , Yang Z , Huang W , Sun H . Effects of atractylodes macrocephala koidz on immune function of mice. J Beihua Univ. 2001;2:122‐124.

[31] Pan Y , Yin D , Zhang N , Xing Y . Effect of a combined treated with Xiaoji decoction about hyperthermia and cisplatin in dealing with cisplatin‐resistant human ovarian cancer cell. Chin Arch Tradit Chin Med. 2008;26:77‐79.

[32] Liu Y , Jia Z , Dong L , Wang R , Qiu G . A randomized pilot study of atractylenolide I on gastric cancer cachexia patients. Evid Based Complement Alternat Med. 2008;5:337‐344. 18830451 10.1093/ecam/nem031 PMC2529387

[33] Li CQ , He LC , Dong HY , Jin JQ . Screening for the anti‐inflammatory activity of fractions and compounds from atractylodes macrocephala koidz. J Ethnopharmacol. 2007;114:212‐217. 17869038 10.1016/j.jep.2007.08.002

[34] Wang CC , Lin SY , Cheng HC , Hou WC . Pro‐oxidant and cytotoxic activities of atractylenolide I in human promyeloleukemic HL‐60 cells. Food Chem Toxicol. 2006;44:1308‐1315. 16624472 10.1016/j.fct.2006.02.008

[35] Wang C , Duan H , He L . Inhibitory effect of atractylenolide I on angiogenesis in chronic inflammation in vivo and in vitro. Eur J Pharmacol. 2009;612:143‐152. 19356732 10.1016/j.ejphar.2009.04.001

[36] Sirchia SM , Tabano S , Monti L , et al. Misbehaviour of XIST RNA in breast cancer cells. PLoS ONE. 2009;4:e5559. 19440381 10.1371/journal.pone.0005559 PMC2679222

[37] Ma L , Zhou Y , Luo X , Gao H , Deng X , Jiang Y . Long non‐coding RNA XIST promotes cell growth and invasion through regulating miR‐497/MACC1 axis in gastric cancer. Oncotarget. 2017;8:4125‐4135. 27911852 10.18632/oncotarget.13670 PMC5354817

[38] Chen DL , Ju HQ , Lu YX , et al. Long non‐coding RNA XIST regulates gastric cancer progression by acting as a molecular sponge of miR‐101 to modulate EZH2 expression. J Exp Clin Cancer Res. 2016;35:142. 27620004 10.1186/s13046-016-0420-1 PMC5020507

[39] Huang KC , Rao PH , Lau CC , et al. Relationship of XIST expression and responses of ovarian cancer to chemotherapy. Mol Cancer Ther. 2002;1:769‐776. 12492109

[40] Xiao Y , Yurievich UA , Yosypovych SV . Long noncoding RNA XIST is a prognostic factor in colorectal cancer and inhibits 5‐fluorouracil‐induced cell cytotoxicity through promoting thymidylate synthase expression. Oncotarget. 2017;8:83171‐83182. 29137332 10.18632/oncotarget.20487 PMC5669958

[41] Zhong M , Bian Z , Wu Z . miR‐30a suppresses cell migration and invasion through downregulation of PIK3CD in colorectal carcinoma. Cell Physiol Biochem. 2013;31:209‐218. 23486085 10.1159/000343362

[42] Liu Z , Tu K , Liu Q . Effects of microRNA‐30a on migration, invasion and prognosis of hepatocellular carcinoma. FEBS Lett. 2014;588:3089‐3097. 24954667 10.1016/j.febslet.2014.06.037

[43] Zeng L , Yu J , Huang T , et al. Differential combinatorial regulatory network analysis related to venous metastasis of hepatocellular carcinoma. BMC Genomics. 2012;13(suppl 8):S14. 10.1186/1471-2164-13-S8-S14 PMC3535701 23282077

[44] Huang QB , Ma X , Zhang X , et al. Down‐regulated miR‐30a in clear cell renal cell carcinoma correlated with tumor hematogenous metastasis by targeting angiogenesis‐specific DLL4. PLoS ONE. 2013;8:e67294. 23826258 10.1371/journal.pone.0067294 PMC3694928

[45] Liu Z , Chen L , Zhang X , et al. RUNX3 regulates vimentin expression via miR‐30a during epithelial‐mesenchymal transition in gastric cancer cells. J Cell Mol Med. 2014;18:610‐623. 24447545 10.1111/jcmm.12209 PMC4000113

[46] Katz B , Reis ST , Viana NI , et al. Comprehensive study of gene and microRNA expression related to epithelial‐mesenchymal transition in prostate cancer. PLoS ONE. 2014;9:e113700. 25409297 10.1371/journal.pone.0113700 PMC4237496

[47] Cheng CW , Wang HW , Chang CW , et al. MicroRNA‐30a inhibits cell migration and invasion by downregulating vimentin expression and is a potential prognostic marker in breast cancer. Breast Cancer Res Treat. 2012;134:1081‐1093. 22476851 10.1007/s10549-012-2034-4

[48] Wang W , Lin H , Zhou L , et al. MicroRNA‐30a‐3p inhibits tumor proliferation, invasiveness and metastasis and is downregulated in hepatocellular carcinoma. Eur J Surg Oncol. 2014;40:1586‐1594. 24290372 10.1016/j.ejso.2013.11.008

[49] Yamaguchi T , Yanagisawa K , Sugiyama R , et al. NKX2‐1/TITF1/TTF‐1‐Induced ROR1 is required to sustain EGFR survival signaling in lung adenocarcinoma. Cancer Cell. 2012;21:348‐361. 22439932 10.1016/j.ccr.2012.02.008

[50] Hojjat‐Farsangi M , Khan AS , Daneshmanesh AH , et al. The tyrosine kinase receptor ROR1 is constitutively phosphorylated in chronic lymphocytic leukemia (CLL) cells. PLoS ONE. 2013;8:e78339. 24205204 10.1371/journal.pone.0078339 PMC3813472

[51] Bicocca VT , Chang BH , Masouleh BK , et al. Crosstalk between ROR1 and the Pre‐B cell receptor promotes survival of t(1;19) acute lymphoblastic leukemia. Cancer Cell. 2012;22:656‐667. 23153538 10.1016/j.ccr.2012.08.027 PMC3500515

[52] O'Connell MP , Marchbank K , Webster MR , et al. Hypoxia induces phenotypic plasticity and therapy resistance in melanoma via the tyrosine kinase receptors ROR1 and ROR2. Cancer Discov. 2013;3:1378‐1393. 24104062 10.1158/2159-8290.CD-13-0005 PMC3918498

[53] Zhang S , Cui B , Lai H , et al. Ovarian cancer stem cells express ROR1, which can be targeted for anti‐cancer‐stem‐cell therapy. Proc Natl Acad Sci USA. 2014;111:17266‐17271. 25411317 10.1073/pnas.1419599111 PMC4260559

[54] Li P , Harris D , Liu Z , Liu J , Keating M , Estrov Z . Stat3 activates the receptor tyrosine kinase like orphan receptor‐1 gene in chronic lymphocytic leukemia cells. PLoS ONE. 2010;5:e11859. 20686606 10.1371/journal.pone.0011859 PMC2912280

[55] Daneshmanesh AH , Hojjat‐Farsangi M , Moshfegh A , et al. The PI3K/AKT/mTOR pathway is involved in direct apoptosis of CLL cells induced by ROR1 monoclonal antibodies. Br J Haematol. 2015;169:455‐458. 25407287 10.1111/bjh.13228

[56] Gentile A , Lazzari L , Benvenuti S , Trusolino L , Comoglio PM . The ROR1 pseudokinase diversifies signaling outputs in MET‐addicted cancer cells. Int J Cancer. 2014;135:2305‐2316. 24706440 10.1002/ijc.28879

[57] Tan H , He Q , Gong G , et al. miR‐382 inhibits migration and invasion by targeting ROR1 through regulating EMT in ovarian cancer. Int J Oncol. 2016;48:181‐190. 26575700 10.3892/ijo.2015.3241

[58] Ye Y , Wang H , Chu JH , et al. Atractylenolide II induces G1 cell‐cycle arrest and apoptosis in B16 melanoma cells. J Ethnopharmacol. 2011;136:279‐282. 21524699 10.1016/j.jep.2011.04.020

[59] Dong H , He L , Huang M , Dong Y . Anti‐inflammatory components isolated from atractylodes macrocephala koidz. Nat Prod Res. 2008;22:1418‐1427. 19023804 10.1080/14786410801931629

